# Strategic Use of Single-Level Vertebral Body Sliding Osteotomy Within a 360-Degree Fusion Construct for the Cervical Ossified Posterior Longitudinal Ligament

**DOI:** 10.7759/cureus.91689

**Published:** 2025-09-05

**Authors:** Linhan Jasmine Ha, Sudhir Suggala, Adnan Shahid, Mehdi Khaleghi, Richard Menger

**Affiliations:** 1 Neurosurgery, University of South Alabama College of Medicine, Mobile, USA; 2 Neurosurgery, University of South Alabama, Mobile, USA

**Keywords:** anterior cervical corpectomy and fusion (accf), anterior cervical discectomy and fusion (acdf), cervical myelopathy, cervical spine kyphosis, ossification of the posterior longitudinal ligament (opll), vertebral body sliding osteotomy (vbso)

## Abstract

Cervical myelopathy caused by ossification of the posterior longitudinal ligament (OPLL) presents significant surgical challenges, especially in multilevel disease with kyphosis or high canal-occupying ratios. While anterior cervical discectomy and fusion (ACDF) and anterior cervical corpectomy and fusion (ACCF) are commonly used, both carry notable risks, particularly in patients with poor bone quality. Vertebral body sliding osteotomy (VBSO) is a novel technique that enables anterior decompression by translating the vertebral body without resection, thereby minimizing dural manipulation and graft-related complications. We present the case of a 48-year-old woman with C4-C7 segmental OPLL, kyphosis, and ventral compression, who underwent staged surgery consisting of ACDF with C5 VBSO followed by posterior decompression and C2-T2 fusion. The patient experienced significant neurological improvement, and imaging confirmed successful decompression and alignment restoration. This case highlights the utility of VBSO in complex cervical pathology and supports its role as a safe and effective alternative to ACCF in appropriately selected patients.

## Introduction

Cervical myelopathy due to ossification of the posterior longitudinal ligament (OPLL) or advanced spondylosis poses a complex surgical problem, particularly when the disease spans multiple levels, is associated with cervical kyphosis, or demonstrates a high canal-occupying ratio [1,2]. Conventional anterior decompression techniques such as anterior cervical discectomy and fusion (ACDF) and anterior cervical corpectomy and fusion (ACCF) are commonly used but not without drawbacks. ACDF may be inadequate when the compressive pathology lies posterior to the vertebral body, and ACCF, though effective, has been associated with increased rates of dural injury, graft complications, and pseudarthrosis, particularly in multilevel constructs [3-6].

Vertebral body sliding osteotomy (VBSO), first described by Lee et al., has emerged as a promising alternative anterior decompression technique [7-9]. By translating the vertebral body anteriorly rather than performing a corpectomy, VBSO achieves effective spinal canal decompression while minimizing manipulation of the OPLL mass and dura interface. The technique allows for improved lordosis restoration, multiple points of anterior fixation, and a shorter biomechanical lever arm, resulting in superior early fusion rates and reduced subsidence compared to ACCF [10,11].

Current literature supports the use of VBSO primarily in cases involving two to three levels, particularly when direct decompression through discectomy is not feasible and when there is rigid kyphosis or a K-line (-) configuration [7,9,10]. For pathology involving more than three levels, the risk of pseudarthrosis and complications such as retropharyngeal hematoma may increase, limiting the application of VBSO without supplemental posterior stabilization [12,13].

We present a case of multilevel cervical myelopathy due to C4-7 segmental OPLL with kyphosis, treated successfully with a staged surgery consisting of C4-5 and C5-6 ACDF with Ames Type 4 osteotomies at C4-5 and C5-6, and a C5 vertebral body sliding osteotomy, followed by C2-T2 fusion and C3-7 decompression. This case illustrates the technical nuances, radiological correction, and clinical improvement achieved through this novel anterior approach, further supporting its use in carefully selected patients and reinforcing its role as a safe and effective alternative to conventional anterior decompression strategies in select patient populations.

## Case presentation

Clinical examination

A 48-year-old woman was referred to our clinic following a motor vehicle collision on September 14, 2024. She reported persistent neck pain and described a progressive decline in hand coordination, including dropping objects and difficulty with fine motor tasks. She also noted gait instability and paresthesias in both upper extremities (C5, C6, C7 dermatomes). Neurological examination revealed a positive Lhermitte’s sign, diminished grip strength (4−/5 on the right, 4/5 on the left), and right upper limb weakness (4−/5), with preserved strength in the remaining left upper and both lower extremities (5/5). Deep tendon reflexes were hyperreflexic; Hoffmann’s sign was positive, and clonus was negative bilaterally. Her past medical history was notable for a cerebrovascular accident during pregnancy in 2006, epilepsy not requiring current medical therapy, and active tobacco use.

Neuroimaging findings

**Figure 1:**
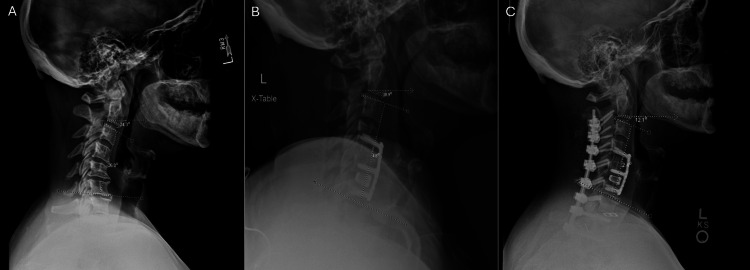
Pre- and postoperative lateral radiographs demonstrating staged correction with improvement in cervical alignment parameters. A 48-year-old woman with cervical spondylotic myelopathy due to segmental ossification of the posterior longitudinal ligament (OPLL) at C4–5, C5–6 (maximum), and C6–7, along with cervical kyphosis, underwent anterior cervical decompression and fusion at C4–5 and C5–6 with vertebral body sliding osteotomy at C5. On preoperative radiographic examination (A), the segmental angle between C3 and C6 was 26° of kyphosis, with a C2 slope of 24.3°. (B) After C4–5 and C5–6 ACDF, the segmental angle between C3 and C6 improved to 4.8° of lordosis, with a C2 slope of 18.9°. (C) Posterior screw fixation was performed due to severe osteoporosis. At two months postoperatively, the segmental lordosis between C3 and C6 was 2.4°, with a C2 slope of 12.1°.

Cervical spine magnetic resonance imaging (MRI) demonstrated disc herniations at C4-5, C5-6, and C6-7 with associated calcification of the posterior longitudinal ligament (PLL) at each level. Additionally, prominent posterior osteophytes were noted at C5-6, contributing to ventral spinal cord compression. Given the multilevel nature of the pathology, the presence of ventral compressive elements not amenable to discectomy alone, and the desire to minimize the risk of dural injury associated with corpectomy, a VBSO was planned (Figures 1, 3F).

Surgical management

**Figure 2:**
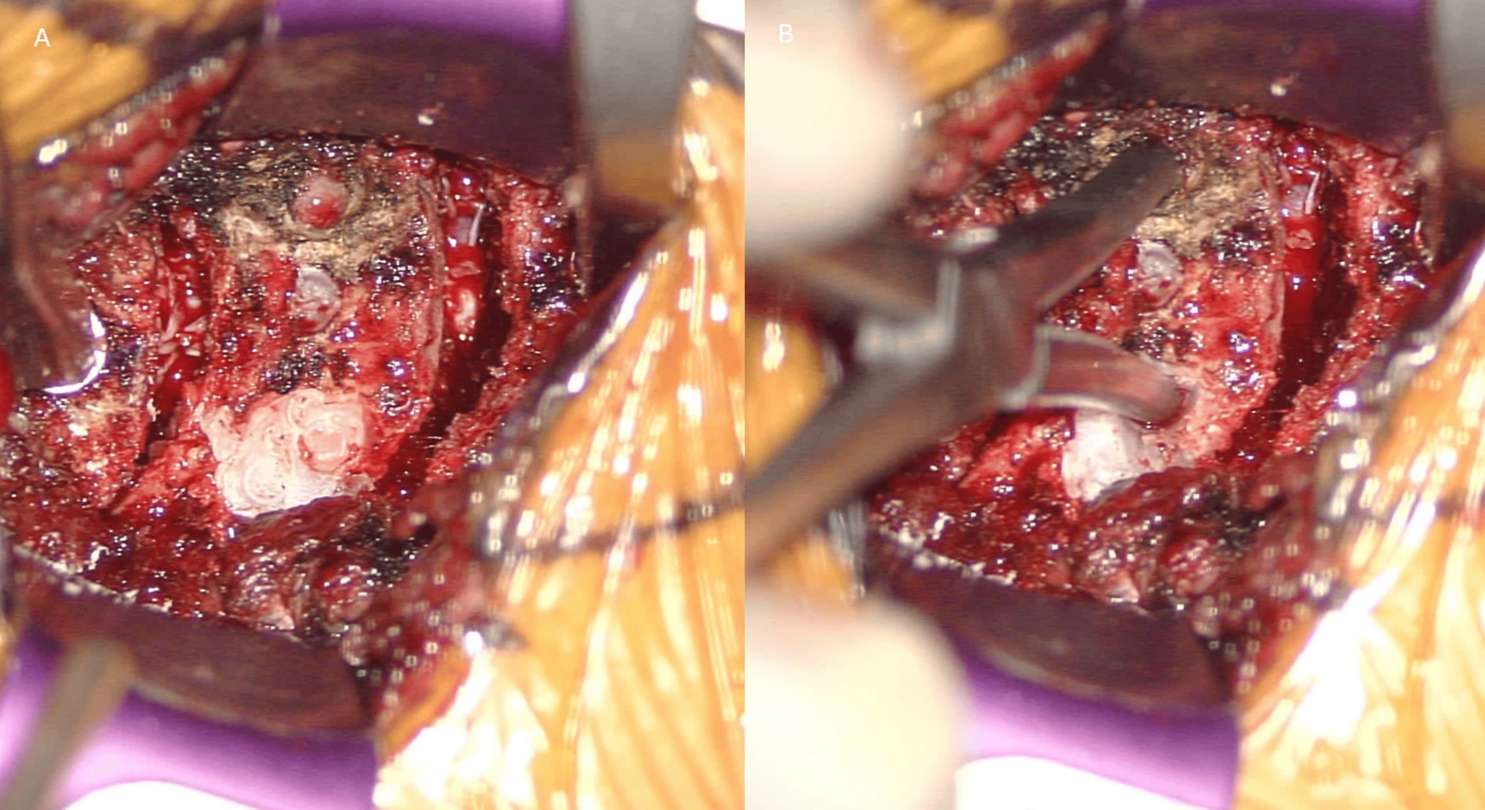
Operative microscope pictures. (A) After performing C4-5 and C5-6 diskectomy (B) After making two parallel longitudinal slits with burr and grasping the mobile fragments using towel clip forceps and pulling them out with a gentle force, interbody cages were inserted and anterior cervical plate affixed to maintain the position.

The patient underwent anterior cervical decompression and fusion at C4-5 and C5-6. A vertebral body sliding osteotomy was performed at the C5 level to achieve anterior translation of the vertebral body along with the associated ossification and osteophytes. Bilateral uncinectomies were performed at both C4-5 and C5-6 to optimize decompression and facilitate mobilization. Interbody cages packed with local autograft were placed, and anterior plating was used to stabilize the construct. The next day, the patient underwent C2-T2 fusion and C3-7 decompression. This was specifically planned as part of a 360-degree fusion and to decompress the C6-7 level indirectly, as this level had ventral compression. The decision not to perform an ACDF at C6-7 and instead address it posteriorly was made due to poor bone quality, which increases the risk of pseudarthrosis (Figure 2).

VBSO procedure

The patient was placed in a supine position with the neck in slight extension. A standard Smith-Robinson anterior cervical approach was used to expose the targeted cervical levels. Discectomies were performed at the superior and inferior levels of the planned osteotomy [7-10,14]. The PLL was resected at these disc levels to permit anterior translation of the vertebral body. Using a 2-3 mm high-speed burr, two longitudinal slits were made along the base of the uncinate processes bilaterally. The posterior cortex of the slits was removed using a Kerrison punch, creating a box-shaped mobile segment. Caspar pins were placed above and below the osteotomy level to provide controlled distraction. The mobilized vertebral body segment was gently pulled anteriorly using Allis forceps, effectively decompressing the spinal canal by translating the OPLL or compressive pathology forward.

Interbody cages packed with local autograft (harvested from endplate osteophytes) were inserted into the disc spaces. These cages helped restore and maintain segmental lordosis and provide structural support. An anterior cervical plate was applied across the construct. Additional anterior translation was achieved by screw tightening through the mobilized vertebral body. The plate was contoured to promote lordosis and prevent loss of reduction.

Postoperative course

**Figure 3:**
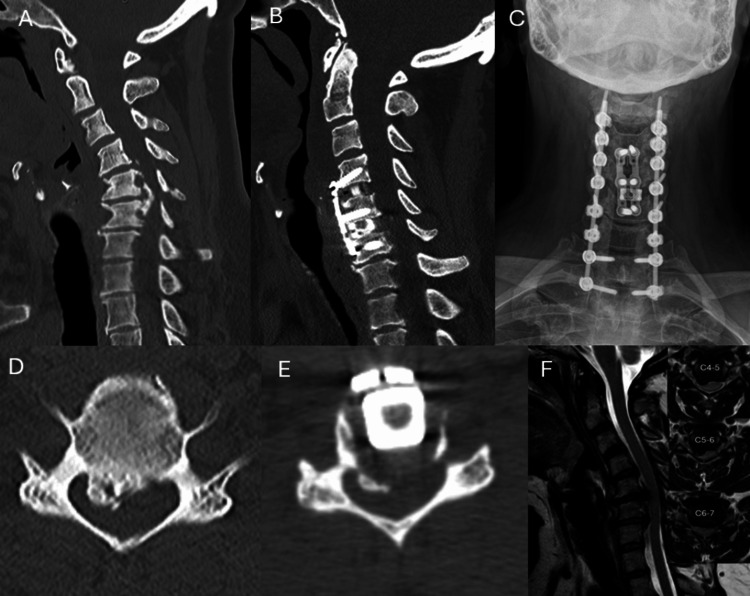
Illustrative case of a 48-year-old woman as shown in Figure 1. Anterior cervical decompression and fusion at C4–5 and C5–6, along with vertebral body sliding osteotomy at C5, were performed. She subsequently underwent posterior cervical decompression laminectomy (C3–7) and C2–T2 fusion. The preoperative (A) and postoperative (B) computed tomography (CT) images and (C) anteroposterior (AP) X-ray demonstrate improved cervical alignment and solid fusion after surgery. The preoperative (D) and postoperative (E) axial images show canal widening achieved by anterior translation of the C5 vertebral body. (F) Preoperative sagittal and axial (inset) magnetic resonance imaging (MRI) scans show cord compression due to cervical OPLL.

The patient tolerated the procedure well without intraoperative complications. Postoperatively, neurological examination revealed improved grip strength (4/5 on the right, 5/5 on the left), but right upper limb weakness (4−/5) persisted in the immediate postoperative period. Follow-up imaging confirmed successful anterior translation of the vertebral body, adequate spinal canal decompression, and appropriate alignment of the cervical spine (Figure 3).

## Discussion

Cervical myelopathy caused by disc herniation, osteophyte formation, and OPLL poses unique challenges, particularly when the pathology spans multiple levels and involves ventral compressive elements not amenable to discectomy alone [9,14,15]. In such cases, ACCF is traditionally considered; however, this approach is associated with increased rates of complications, including dural tears, graft subsidence, and pseudarthrosis, particularly when the OPLL mass is adherent to the dura or when multiple levels are involved [10,11,16,17].

VBSO offers a novel anterior approach that allows for effective ventral decompression by translating the vertebral body anteriorly, thereby mobilizing the compressive mass without direct manipulation of the dura. This technique avoids corpectomy while preserving endplate integrity, creating a construct with multiple fixation points and a shorter biomechanical lever arm compared to ACCF. In a comparative study, Lee et al. reported significantly higher one-year fusion rates and lower subsidence with VBSO compared to both ACDF and ACCF, along with a comparable complication profile.

Our patient underwent anterior cervical decompression and fusion at C4-5 and C5-6. A vertebral body sliding osteotomy was performed at the C5 level to achieve anterior translation of the vertebral body and associated ossified and osteophytic elements, enabling effective decompression of the spinal canal. Bilateral uncinectomies were performed at both C4-5 and C5-6 to facilitate mobilization and optimize neural decompression. Interbody cages packed with local autograft were inserted at both levels, and an anterior cervical plate was applied to provide immediate construct stability.

On postoperative day one, the patient underwent a planned second-stage posterior surgery consisting of C3-7 decompression and instrumented fusion from C2 to T2. This was performed to complete a 360-degree circumferential fusion and to provide indirect decompression of the C6-7 level, where ventral compression had been noted on imaging. A decision was made to defer ACDF at C6-7 due to the patient’s active smoking status, history of prior stroke, and suboptimal bone quality, factors that significantly increase the risk of pseudarthrosis following anterior-only constructs. Posterior stabilization was thus preferred to ensure robust biomechanical support and improve the likelihood of achieving solid fusion.

Notably, Lee et al. have recommended VBSO primarily for two to three levels of involvement, especially when sagittal alignment is unfavorable or in K-line (-) patients, where posterior decompression is less effective [8]. Although our patient’s myelopathy was precipitated by trauma, her imaging and clinical signs were consistent with underlying degenerative and ossified disease that had likely been subclinical until exacerbated by the collision. The use of VBSO at a single vertebral level (C5) in this context allowed for successful decompression and symptom resolution without resorting to a more extensive corpectomy.

The patient’s postoperative course was uneventful, and her neurological symptoms improved significantly, consistent with prior reports showing favorable functional recovery following VBSO. The fusion construct was stable, with radiographic evidence of appropriate anterior translation and restoration of cervical alignment.

This case is unique in demonstrating the selective use of VBSO at a single vertebral level (C5) in a patient presenting with post-traumatic cervical myelopathy on a background of multilevel degenerative and ossified pathology [12,13]. Unlike prior reports focused primarily on VBSO for chronic OPLL or fixed kyphotic deformity, our patient presented acutely following a motor vehicle collision, highlighting the utility of VBSO in an acute-on-chronic scenario. Furthermore, the procedure was integrated into a two-stage circumferential (360-degree) fusion strategy, with anterior decompression and VBSO followed by posterior decompression and C2-T2 instrumentation. This approach allowed indirect decompression of the C6-7 level while minimizing the risk of pseudarthrosis in a high-risk patient with poor bone quality and active tobacco use. The case underscores the adaptability of VBSO when used judiciously in hybrid constructs and the importance of personalized surgical planning in complex cervical spine pathology.

VBSO, though effective for anterior decompression in cervical myelopathy with kyphotic deformity or OPLL, has several limitations. It is a technically demanding procedure with a steep learning curve, requiring precise osteotomy and controlled anterior translation of the vertebral body, which increases the risk of complications in inexperienced hands. VBSO is not ideal in patients with severe osteoporosis or posterior pathologies and is limited in cases of multilevel disease with significant disc degeneration or global ankylosis. Long-term outcome data are sparse, with most studies being small, single-center series, and concerns remain regarding residual kyphosis, incomplete deformity correction, and the potential for adjacent segment degeneration.

## Conclusions

This case highlights the effective use of VBSO as a targeted anterior decompression technique in the management of multilevel cervical myelopathy with central ossified and bony pathology. In a patient with disc herniations, posterior osteophytes, and calcification of the PLL, VBSO allowed safe anterior translation of compressive elements while preserving vertebral body integrity and avoiding the morbidity associated with corpectomy. When used in conjunction with ACDF, VBSO provides a valuable alternative for managing complex ventral cervical spine compression, particularly in cases involving one to three levels where standard techniques may fall short. This case adds to the growing body of evidence supporting VBSO as a safe and effective approach in carefully selected patients.
